# Supplementary material for: Hot spot prediction in protein-protein interactions by an ensemble system
Source: BMC Syst Biol. 2018 Dec 31;12(Suppl 9):132. doi: 10.1186/s12918-018-0665-8 (PMC6311905; doi:10.1186/s12918-018-0665-8)
Supplement: Supplementary file 2 — All relevant MATLAB code. A simple Matlab implement of our predictor. (ZIP 19,742 kb) [file 12918_2018_665_MOESM2_ESM.zip › Additional file 2/Read ASA/ASA website/dbMPIKT/3NGBB.html]

 NetSurfP - Protein Surface Accessibility and Secondary Structure Predictions

  

|  |  |  |
| --- | --- | --- |
|  |  | NetSurfP - Protein Surface Accessibility and Secondary Structure Predictions  Technical University of Denmark |

  


---

```
# For publication of results, please cite:
# A generic method for assignment of reliability scores applied to solvent accessibility predictions.
# Bent Petersen, Thomas Nordahl Petersen, Pernille Andersen, Morten Nielsen and Claus Lundegaard
# BMC Structural Biology 2009, 9:51 doi:10.1186/1472-6807-9-51
#
# Column 1: Class assignment - B for buried or E for Exposed - Threshold: 25% exposure, but not based on RSA
# Column 2: Amino acid
# Column 3: Sequence name
# Column 4: Amino acid number
# Column 5: Relative Surface Accessibility - RSA
# Column 6: Absolute Surface Accessibility
# Column 7: Z-fit score for RSA prediction
# Column 8: Probability for Alpha-Helix
# Column 9: Probability for Beta-strand
# Column 10: Probability for Coil
E Q  3NGBB                  1    0.744 132.843   1.180   0.005   0.015   0.979
B V  3NGBB                  2    0.209  32.200  -0.175   0.003   0.718   0.279
E Q  3NGBB                  3    0.428  76.459   1.229   0.001   0.900   0.099
B L  3NGBB                  4    0.155  28.454   0.829   0.002   0.816   0.182
E V  3NGBB                  5    0.387  59.559   0.519   0.021   0.756   0.223
B Q  3NGBB                  6    0.239  42.685   0.679   0.004   0.514   0.481
E S  3NGBB                  7    0.429  50.279  -0.533   0.020   0.205   0.775
E G  3NGBB                  8    0.497  39.098  -1.857   0.018   0.088   0.893
E G  3NGBB                  9    0.520  40.948  -2.267   0.018   0.047   0.935
E Q  3NGBB                 10    0.489  87.371  -1.636   0.020   0.205   0.775
E M  3NGBB                 11    0.399  79.760  -0.842   0.021   0.451   0.528
E K  3NGBB                 12    0.300  61.813   0.647   0.021   0.451   0.528
E K  3NGBB                 13    0.414  85.078   1.259   0.021   0.279   0.699
E P  3NGBB                 14    0.446  63.231  -0.472   0.018   0.047   0.935
E G  3NGBB                 15    0.606  47.669  -1.982   0.005   0.045   0.951
E E  3NGBB                 16    0.428  74.841  -0.620   0.004   0.138   0.858
E S  3NGBB                 17    0.430  50.349  -0.472   0.004   0.420   0.576
B M  3NGBB                 18    0.153  30.655   0.811   0.002   0.816   0.182
E R  3NGBB                 19    0.408  93.546   0.879   0.001   0.900   0.099
B I  3NGBB                 20    0.073  13.561   0.328   0.001   0.900   0.099
B S  3NGBB                 21    0.169  19.830   0.683   0.001   0.900   0.099
B C  3NGBB                 22    0.042   5.911   0.221   0.001   0.900   0.099
B R  3NGBB                 23    0.287  65.792   0.522   0.001   0.900   0.099
B A  3NGBB                 24    0.095  10.469  -0.548   0.002   0.816   0.182
B S  3NGBB                 25    0.173  20.276  -0.542   0.004   0.514   0.481
B G  3NGBB                 26    0.241  18.935  -1.857   0.004   0.197   0.799
B Y  3NGBB                 27    0.240  51.224  -0.991   0.005   0.336   0.660
B E  3NGBB                 28    0.197  34.416  -0.533   0.004   0.514   0.481
B F  3NGBB                 29    0.103  20.732  -1.014   0.021   0.451   0.528
B I  3NGBB                 30    0.223  41.329  -1.159   0.021   0.279   0.699
B D  3NGBB                 31    0.299  43.071  -1.174   0.018   0.088   0.893
B C  3NGBB                 32    0.106  14.840  -0.005   0.019   0.141   0.840
B T  3NGBB                 33    0.084  11.692   0.313   0.022   0.359   0.619
B L  3NGBB                 34    0.029   5.347   0.241   0.021   0.756   0.223
B N  3NGBB                 35    0.050   7.305   0.141   0.021   0.756   0.223
B W  3NGBB                 36    0.036   8.658   0.436   0.021   0.756   0.223
B I  3NGBB                 37    0.037   6.845   0.236   0.018   0.846   0.136
B R  3NGBB                 38    0.125  28.671   0.452   0.023   0.655   0.322
B L  3NGBB                 39    0.091  16.589   0.365   0.022   0.359   0.619
B A  3NGBB                 40    0.202  22.216   0.858   0.005   0.045   0.951
E P  3NGBB                 41    0.349  49.537   0.724   0.005   0.015   0.979
E G  3NGBB                 42    0.562  44.206  -1.568   0.018   0.019   0.964
E K  3NGBB                 43    0.397  81.642   0.661   0.018   0.019   0.964
E R  3NGBB                 44    0.423  96.821  -0.454   0.018   0.088   0.893
B P  3NGBB                 45    0.159  22.619   0.990   0.005   0.262   0.733
B E  3NGBB                 46    0.087  15.269  -0.259   0.004   0.616   0.381
B W  3NGBB                 47    0.048  11.472   0.077   0.001   0.900   0.099
B M  3NGBB                 48    0.024   4.882   0.877   0.001   0.959   0.040
B G  3NGBB                 49    0.018   1.377  -0.144   0.001   0.959   0.040
B W  3NGBB                 50    0.078  18.711  -0.400   0.001   0.959   0.040
B L  3NGBB                 51    0.023   4.120   0.902   0.001   0.959   0.040
B K  3NGBB                 52    0.155  31.986   0.616   0.002   0.816   0.182
B P  3NGBB                 53    0.231  32.850  -1.070   0.005   0.336   0.660
E R  3NGBB                 54    0.582 133.278  -1.895   0.005   0.015   0.979
E G  3NGBB                 55    0.594  46.724  -2.697   0.003   0.003   0.994
E G  3NGBB                 56    0.535  42.097  -2.319   0.018   0.019   0.964
E A  3NGBB                 57    0.454  50.020  -0.672   0.018   0.047   0.935
E V  3NGBB                 58    0.340  52.212  -0.219   0.020   0.205   0.775
E N  3NGBB                 59    0.438  64.109  -0.041   0.020   0.205   0.775
B Y  3NGBB                 60    0.215  45.967   0.357   0.064   0.216   0.721
B A  3NGBB                 61    0.201  22.172  -0.278   0.052   0.084   0.864
E R  3NGBB                 62    0.599 137.171  -0.710   0.053   0.043   0.903
E P  3NGBB                 63    0.383  54.362  -0.026   0.052   0.084   0.864
B L  3NGBB                 64    0.079  14.392  -0.436   0.020   0.205   0.775
E Q  3NGBB                 65    0.363  64.760   0.807   0.021   0.279   0.699
B G  3NGBB                 66    0.183  14.363  -1.918   0.004   0.420   0.576
B R  3NGBB                 67    0.195  44.678   0.514   0.002   0.816   0.182
B V  3NGBB                 68    0.047   7.270  -0.098   0.001   0.900   0.099
B T  3NGBB                 69    0.128  17.712   0.956   0.001   0.959   0.040
B M  3NGBB                 70    0.043   8.644   0.608   0.001   0.959   0.040
B T  3NGBB                 71    0.107  14.785   0.360   0.002   0.816   0.182
E R  3NGBB                 72    0.265  60.708   0.828   0.004   0.616   0.381
B D  3NGBB                 73    0.237  34.209  -0.485   0.004   0.420   0.576
E V  3NGBB                 74    0.428  65.737  -0.168   0.018   0.088   0.893
E Y  3NGBB                 75    0.320  68.299  -1.907   0.018   0.047   0.935
E S  3NGBB                 76    0.512  59.983   0.645   0.019   0.141   0.840
B D  3NGBB                 77    0.239  34.397  -0.417   0.022   0.359   0.619
B T  3NGBB                 78    0.176  24.411   1.098   0.018   0.846   0.136
B A  3NGBB                 79    0.061   6.689  -0.325   0.011   0.918   0.071
B F  3NGBB                 80    0.151  30.225   0.646   0.006   0.962   0.032
B L  3NGBB                 81    0.042   7.635   0.871   0.001   0.959   0.040
B E  3NGBB                 82    0.135  23.602  -0.093   0.001   0.900   0.099
B L  3NGBB                 83    0.068  12.451  -0.560   0.003   0.718   0.279
E R  3NGBB                 84    0.347  79.394  -0.153   0.004   0.420   0.576
E S  3NGBB                 85    0.388  45.474  -0.787   0.004   0.197   0.799
B L  3NGBB                 86    0.127  23.235  -0.041   0.004   0.085   0.910
E T  3NGBB                 87    0.483  66.950   1.065   0.018   0.047   0.935
E V  3NGBB                 88    0.388  59.574  -0.772   0.052   0.084   0.864
E D  3NGBB                 89    0.461  66.372  -0.677   0.053   0.043   0.903
B D  3NGBB                 90    0.173  24.915  -0.666   0.052   0.084   0.864
B T  3NGBB                 91    0.106  14.716   0.381   0.069   0.386   0.545
B A  3NGBB                 92    0.033   3.637  -0.309   0.018   0.846   0.136
B V  3NGBB                 93    0.058   8.945   1.008   0.011   0.918   0.071
B Y  3NGBB                 94    0.028   5.962   1.145   0.011   0.918   0.071
B F  3NGBB                 95    0.047   9.373   0.964   0.018   0.846   0.136
B C  3NGBB                 96    0.014   1.980   1.604   0.021   0.756   0.223
B T  3NGBB                 97    0.027   3.814   0.162   0.023   0.655   0.322
B R  3NGBB                 98    0.107  24.572   0.084   0.021   0.451   0.528
B G  3NGBB                 99    0.205  16.165  -0.414   0.020   0.205   0.775
B K  3NGBB                100    0.288  59.200  -0.920   0.018   0.088   0.893
E N  3NGBB                101    0.403  58.926  -2.316   0.018   0.047   0.935
E C  3NGBB                102    0.350  49.140  -2.153   0.018   0.047   0.935
E D  3NGBB                103    0.318  45.838  -2.183   0.018   0.047   0.935
E Y  3NGBB                104    0.332  70.884  -1.439   0.018   0.047   0.935
E N  3NGBB                105    0.339  49.674  -1.872   0.018   0.047   0.935
B W  3NGBB                106    0.265  63.732  -0.731   0.018   0.047   0.935
B D  3NGBB                107    0.117  16.903  -0.989   0.018   0.088   0.893
B F  3NGBB                108    0.055  10.998  -0.016   0.019   0.141   0.840
B E  3NGBB                109    0.084  14.622  -0.762   0.020   0.205   0.775
B H  3NGBB                110    0.065  11.805  -0.330   0.020   0.205   0.775
B W  3NGBB                111    0.041   9.788  -0.591   0.019   0.141   0.840
B G  3NGBB                112    0.037   2.943  -1.971   0.019   0.141   0.840
B R  3NGBB                113    0.177  40.419  -0.187   0.004   0.085   0.910
B G  3NGBB                114    0.134  10.546  -1.402   0.004   0.197   0.799
B T  3NGBB                115    0.080  11.054  -0.119   0.004   0.420   0.576
E P  3NGBB                116    0.287  40.754   0.025   0.003   0.718   0.279
B V  3NGBB                117    0.048   7.454   0.562   0.001   0.900   0.099
B I  3NGBB                118    0.133  24.623  -0.036   0.002   0.816   0.182
B V  3NGBB                119    0.068  10.375   0.194   0.002   0.816   0.182
B S  3NGBB                120    0.165  19.315  -0.639   0.004   0.514   0.481
B S  3NGBB                121    0.177  20.698  -1.169   0.020   0.205   0.775
E P  3NGBB                122    0.437  61.939  -1.446   0.018   0.047   0.935
E S  3NGBB                123    0.455  53.361  -0.738   0.018   0.047   0.935
E T  3NGBB                124    0.516  71.583  -1.598   0.018   0.047   0.935
E K  3NGBB                125    0.602 123.790  -1.593   0.005   0.015   0.979
E G  3NGBB                126    0.485  38.185  -1.757   0.005   0.015   0.979
B P  3NGBB                127    0.350  49.594  -1.745   0.005   0.045   0.951
E S  3NGBB                128    0.515  60.323  -0.941   0.004   0.197   0.799
B V  3NGBB                129    0.284  43.605  -1.658   0.005   0.336   0.660
B F  3NGBB                130    0.150  30.045   0.149   0.005   0.336   0.660
E P  3NGBB                131    0.280  39.732  -0.802   0.005   0.262   0.733
B L  3NGBB                132    0.104  18.987  -1.121   0.005   0.262   0.733
E A  3NGBB                133    0.296  32.652  -0.988   0.004   0.197   0.799
B P  3NGBB                134    0.261  36.979  -1.623   0.004   0.085   0.910
B S  3NGBB                135    0.160  18.705  -2.088   0.004   0.085   0.910
E S  3NGBB                136    0.458  53.642  -2.252   0.005   0.045   0.951
E K  3NGBB                137    0.720 148.104  -2.437   0.005   0.015   0.979
E S  3NGBB                138    0.344  40.340  -2.676   0.005   0.015   0.979
E T  3NGBB                139    0.508  70.487  -1.491   0.005   0.015   0.979
E S  3NGBB                140    0.521  61.049  -1.036   0.005   0.015   0.979
E G  3NGBB                141    0.630  49.550  -2.719   0.003   0.003   0.994
E G  3NGBB                142    0.518  40.798  -1.854   0.005   0.015   0.979
E T  3NGBB                143    0.417  57.782   0.323   0.005   0.262   0.733
B A  3NGBB                144    0.076   8.386   0.543   0.002   0.816   0.182
B A  3NGBB                145    0.223  24.597   0.700   0.001   0.900   0.099
B L  3NGBB                146    0.053   9.704   0.183   0.001   0.959   0.040
B G  3NGBB                147    0.058   4.588  -0.056   0.001   0.900   0.099
B C  3NGBB                148    0.027   3.819   0.240   0.001   0.900   0.099
B L  3NGBB                149    0.161  29.461   0.692   0.001   0.900   0.099
B V  3NGBB                150    0.043   6.640  -0.254   0.002   0.816   0.182
E K  3NGBB                151    0.299  61.484   0.205   0.004   0.420   0.576
E D  3NGBB                152    0.451  65.032  -1.767   0.004   0.138   0.858
B Y  3NGBB                153    0.222  47.399  -0.658   0.004   0.197   0.799
E F  3NGBB                154    0.335  67.315  -0.182   0.004   0.197   0.799
B P  3NGBB                155    0.197  27.997  -1.210   0.004   0.197   0.799
E E  3NGBB                156    0.597 104.243  -1.012   0.004   0.138   0.858
E P  3NGBB                157    0.406  57.540  -1.124   0.005   0.262   0.733
B V  3NGBB                158    0.147  22.671   0.239   0.003   0.718   0.279
B T  3NGBB                159    0.311  43.122  -0.314   0.001   0.900   0.099
B V  3NGBB                160    0.063   9.652   0.068   0.001   0.959   0.040
B S  3NGBB                161    0.227  26.604   0.340   0.001   0.900   0.099
B W  3NGBB                162    0.072  17.436  -0.359   0.011   0.918   0.071
E N  3NGBB                163    0.341  49.952   0.144   0.003   0.718   0.279
E S  3NGBB                164    0.495  58.073  -0.580   0.005   0.336   0.660
E G  3NGBB                165    0.498  39.200  -1.024   0.018   0.088   0.893
E A  3NGBB                166    0.577  63.596  -0.366   0.018   0.019   0.964
E L  3NGBB                167    0.512  93.692  -1.047   0.018   0.047   0.935
E T  3NGBB                168    0.537  74.510  -1.996   0.018   0.088   0.893
E S  3NGBB                169    0.570  66.851  -0.257   0.004   0.085   0.910
E G  3NGBB                170    0.416  32.739  -0.558   0.004   0.138   0.858
B V  3NGBB                171    0.168  25.791  -0.456   0.004   0.420   0.576
E H  3NGBB                172    0.329  59.772   0.319   0.002   0.816   0.182
B T  3NGBB                173    0.252  34.897  -0.346   0.001   0.900   0.099
B F  3NGBB                174    0.144  28.981   1.170   0.002   0.816   0.182
B P  3NGBB                175    0.151  21.455  -0.620   0.022   0.552   0.426
B A  3NGBB                176    0.223  24.542  -1.149   0.004   0.616   0.381
B V  3NGBB                177    0.182  28.004  -1.751   0.002   0.816   0.182
E L  3NGBB                178    0.316  57.878  -0.365   0.004   0.616   0.381
E Q  3NGBB                179    0.461  82.352  -0.050   0.005   0.336   0.660
E S  3NGBB                180    0.713  83.587  -1.590   0.004   0.085   0.910
E S  3NGBB                181    0.737  86.400  -1.662   0.005   0.015   0.979
E G  3NGBB                182    0.430  33.810  -1.502   0.004   0.085   0.910
E L  3NGBB                183    0.350  64.103   0.533   0.004   0.616   0.381
B Y  3NGBB                184    0.146  31.093   0.023   0.001   0.900   0.099
B S  3NGBB                185    0.156  18.307  -0.701   0.001   0.900   0.099
B L  3NGBB                186    0.094  17.248  -0.617   0.001   0.959   0.040
B S  3NGBB                187    0.151  17.732  -0.374   0.001   0.959   0.040
B S  3NGBB                188    0.050   5.883  -1.044   0.001   0.959   0.040
B V  3NGBB                189    0.203  31.124   0.010   0.001   0.959   0.040
B V  3NGBB                190    0.052   7.916   0.092   0.001   0.959   0.040
E T  3NGBB                191    0.293  40.653   0.861   0.001   0.900   0.099
B V  3NGBB                192    0.069  10.636   0.949   0.002   0.816   0.182
E P  3NGBB                193    0.369  52.389   1.112   0.022   0.552   0.426
E S  3NGBB                194    0.389  45.603  -0.257   0.021   0.279   0.699
E S  3NGBB                195    0.614  72.019  -0.660   0.022   0.359   0.619
E S  3NGBB                196    0.619  72.582   0.340   0.021   0.279   0.699
B L  3NGBB                197    0.214  39.257  -0.293   0.020   0.205   0.775
E G  3NGBB                198    0.707  55.680   0.234   0.005   0.045   0.951
E T  3NGBB                199    0.734 101.820   0.829   0.005   0.015   0.979
E Q  3NGBB                200    0.616 110.107   0.840   0.004   0.197   0.799
E T  3NGBB                201    0.526  73.012   1.188   0.003   0.718   0.279
B Y  3NGBB                202    0.074  15.878   0.626   0.001   0.959   0.040
E I  3NGBB                203    0.375  69.375   1.860   0.001   0.959   0.040
B C  3NGBB                204    0.025   3.510   0.119   0.001   0.959   0.040
E N  3NGBB                205    0.383  56.130   1.091   0.001   0.900   0.099
B V  3NGBB                206    0.074  11.328  -0.757   0.002   0.816   0.182
E N  3NGBB                207    0.560  81.955   0.628   0.002   0.816   0.182
B H  3NGBB                208    0.176  31.996   0.008   0.004   0.514   0.481
E K  3NGBB                209    0.605 124.407   0.261   0.005   0.045   0.951
E P  3NGBB                210    0.695  98.635  -1.632   0.005   0.015   0.979
E S  3NGBB                211    0.564  66.136  -0.640   0.004   0.085   0.910
E N  3NGBB                212    0.580  84.971  -0.030   0.004   0.085   0.910
E T  3NGBB                213    0.467  64.759   0.611   0.005   0.336   0.660
E K  3NGBB                214    0.564 115.953   1.525   0.003   0.718   0.279
B V  3NGBB                215    0.136  20.857   0.395   0.002   0.816   0.182
E D  3NGBB                216    0.651  93.838  -0.077   0.001   0.900   0.099
E K  3NGBB                217    0.305  62.821   1.196   0.002   0.816   0.182
E K  3NGBB                218    0.535 109.988   0.609   0.002   0.816   0.182
E A  3NGBB                219    0.254  28.002  -0.092   0.004   0.616   0.381
E E  3NGBB                220    0.522  91.246   0.011   0.005   0.336   0.660
E P  3NGBB                221    0.505  71.603  -0.426   0.004   0.138   0.858
E K  3NGBB                222    0.629 129.365  -0.138   0.018   0.088   0.893
E S  3NGBB                223    0.592  69.359  -0.273   0.018   0.047   0.935
E C  3NGBB                224    0.590  82.794  -1.978   0.000   0.000   0.999

Explain the output.  Go back.
```

---
